# Engaging chromatin: PRC2 structure meets function

**DOI:** 10.1038/s41416-019-0615-2

**Published:** 2019-11-11

**Authors:** Paul Chammas, Ivano Mocavini, Luciano Di Croce

**Affiliations:** 1 Centre for Genomic Regulation (CRG), The Barcelona Institute of Science and Technology (BIST), Dr. Aiguader 88, Barcelona, 08003 Spain; 2 Universitat Pompeu Fabra (UPF), Barcelona, Spain; 3 ICREA, Pg Lluis Companys 23, Barcelona, 08010 Spain

**Keywords:** Epigenetics, Cancer

## Abstract

Polycomb repressive complex 2 (PRC2) is a key epigenetic multiprotein complex involved in the regulation of gene expression in metazoans. PRC2 is formed by a tetrameric core that endows the complex with histone methyltransferase activity, allowing it to mono-, di- and tri-methylate histone H3 on lysine 27 (H3K27me1/2/3); H3K27me3 is a hallmark of facultative heterochromatin. The core complex of PRC2 is bound by several associated factors that are responsible for modulating its targeting specificity and enzymatic activity. Depletion and/or mutation of the subunits of this complex can result in severe developmental defects, or even lethality. Furthermore, mutations of these proteins in somatic cells can be drivers of tumorigenesis, by altering the transcriptional regulation of key tumour suppressors or oncogenes. In this review, we present the latest results from structural studies that have characterised PRC2 composition and function. We compare this information with data and literature for both gain-of function and loss-of-function missense mutations in cancers to provide an overview of the impact of these mutations on PRC2 activity.

## Background

Transcriptional diversity is one of the hallmarks of cellular identity. It is largely regulated at the level of chromatin, where different protein complexes act as initiators, enhancers and/or repressors of transcription. Among these complexes, are epigenetic modifiers, which are able to catalyse post-translational modifications (PTMs)—such as methylation, acetylation, phosphorylation or ubiquitination—of histone proteins. These modifications can influence gene expression by modulating chromatin accessibility, its interaction with other proteins and its three-dimensional organisation. The Polycomb group (PcG) proteins form histone-modifying complexes whose activity is associated with transcriptional silencing of facultative heterochromatin.^1–4^ Two catalytically distinct complexes can be distinguished: the Polycomb repressive complex (PRC) 1, and PRC2. PRC1 catalyses the mono-ubiquitination of lysine 119 on histone H2A (H2AK119ub),^5,6^ whereas PRC2 catalyses the mono-, di- and tri-methylation of lysine 27 on the histone H3 tail (H3K27me1/2/3).^7^ In mice and humans, PRC2 is essential for proper embryonic stem cell (ESC) fate specification, as it regulates the expression of key developmental genes.^8–10^ Indeed, depletion of PRC2 subunits leads to severe developmental defects with early embryonic or perinatal lethality.^10–17^ Mutations and/or dysregulation of PcG genes are found in several cancer types, especially haematological ones,^18,19^ as well as in rare genetic diseases associated with overgrowth, such as Weaver syndrome.^20^ These alterations can affect PRC2 recruitment and enzymatic activity, leading to changes in the expression of tumour suppressors or oncogenes.

The PRC2 core comprises three stoichiometric factors: enhancer of Zeste (EZH)1 or EZH2, which has a SET domain and is the catalytic subunit of the complex;^7,21^ suppressor of Zeste (SUZ) 12; and embryonic ectoderm development (EED) (Table 1). These three proteins form the minimal core that confers histone methyltransferase (HMT) activity. A fourth factor, retinoblastoma-binding protein (RBBP)4/7 (also known as RBAP48/46), has a slightly lower stoichiometry and is dispensable for the enzymatic activity of the complex.^22,23^ Although there is very little diversity in PRC2 core components, a large number of facultative subunits have been shown to bind PRC2 in a sub-stoichiometric and cell-type specific manner,^24–26^ adding both to the complexity of recruitment of this complex to chromatin and to additional possibilities of regulation of its enzymatic activity.^27^ Studies carried out over the past 5 years have shown that many of these facultative subunits bind in a mutually exclusive manner, giving rise to two versions of the PRC2 complex. The first variant (PRC2.1) comprises one of three Polycomb-like (PCL) proteins (PCL1/2/3, also named PHF1, MTF2 and PHF19, respectively) as well as Elongin BC and Polycomb repressive complex 2-associated protein (EPOP) or PRC2-associated LCOR isoform 1 (PALI1/2), while the other variant (PRC2.2) comprises Jumonji and AT-rich interaction domain 2 (JARID2) and adipocyte enhancer-binding protein 2 (AEBP2),^26,28–30^ in addition to the core components.

**Table 1:** Domain composition of PRC2 subunits

Protein	Name	Acronym
SUZ12	Zn finger binding domain	ZnB
	WD-domain binding 1	WDB1
	C2 domain	C2
	Zn Finger	Zn
	WD-domain binding 2	WDB2
	VRN2-EMF2-FIS2-Su(z)12 box	VEFS
EZH2	SANT1L-binding domain	SBD
	EED-binding domain	EBD
	β-addition motif	BAM
	SET activation loop	SAL
	stimulation-responsive motif	SRM
	Swi3, Ada2, N-CoR and TFIIIB DNA-binding domain 1 like	SANT1
	Motif connecting SANT1 and SANT2	MCSS
	SANT2-like	SANT2
	CXC domain	CXC
	Su(var)3-9, E(z) and Trx domain	SET
	Post-SET	Post-SET
EED	WD-repeat region	WD1
		WD2
		WD3
		WD4
		WD5
		WD6
		WD7
PALI1	Nuclear receptor binding box	NR
	CTBP binding motifs (x2)	CTBP
	G9A interaction region	
	Pali interaction with PRC2 domain	PIP
RBBP4	WD-repeat region	WD1
		WD2
		WD3
		WD4
		WD5
		WD6
		WD7
RBBP7	WD-repeat region	WD1
		WD2
		WD3
		WD4
		WD5
		WD6
		WD7
PHF1/PCL1	Tudor domain	Tudor
	PHD Domain	PHD1
	PHD Domain	PHD2
	Extended Homology domain	EH
	Chromo domain	Chromo
MTF2/PCL2	Tudor domain	Tudor
	PHD Domain	PHD1
	PHD Domain	PHD2
	Extended Homology domain	EH
	Chromo domain	Chromo
PHF19/PCL3	Tudor domain	Tudor
	PHD Domain	PHD1
	PHD Domain	PHD2
	Extended Homology domain	EH
	Chromo domain	Chromo
EPOP	ELOBC binding box	BC box
	C-terminal region	CTR
AEBP2	Zn finger	Zn1
	Zn finger	Zn2
	Zn finger	Zn3
	Lysine/Arginine-rich domain	KR
	C2 binding domain	C2B
	H3K4 displacement domain	H3K4D
JARID2	Transrepression domain	TR
	Ezh1/2-binding domain	
	Nucleosome interaction domain	
	Jumonji N-term	JmjN
	AT-rich interaction domain	ARID
	Jumonji C-term	JmjC
	Zinc finger	ZF

Along with the interest in characterising the functional role of accessory factors in regulating PRC2 activity, effort has also been put into trying to gain structural insights into the complexity of PRC2 and its subtypes. In this review, we discuss the latest findings regarding the PRC2 structure, focusing on the aspects that define the formation of different complex subtypes, its chromatin targeting and its enzymatic activity. Finally, building on all the current structural knowledge, we highlight the potential effect of PRC2 mutations on complex integrity and activity, and the role of mutations of PRC2 components in cancer.

## Structural basis for PRC2 complex formation and function

**Fig. 1:**
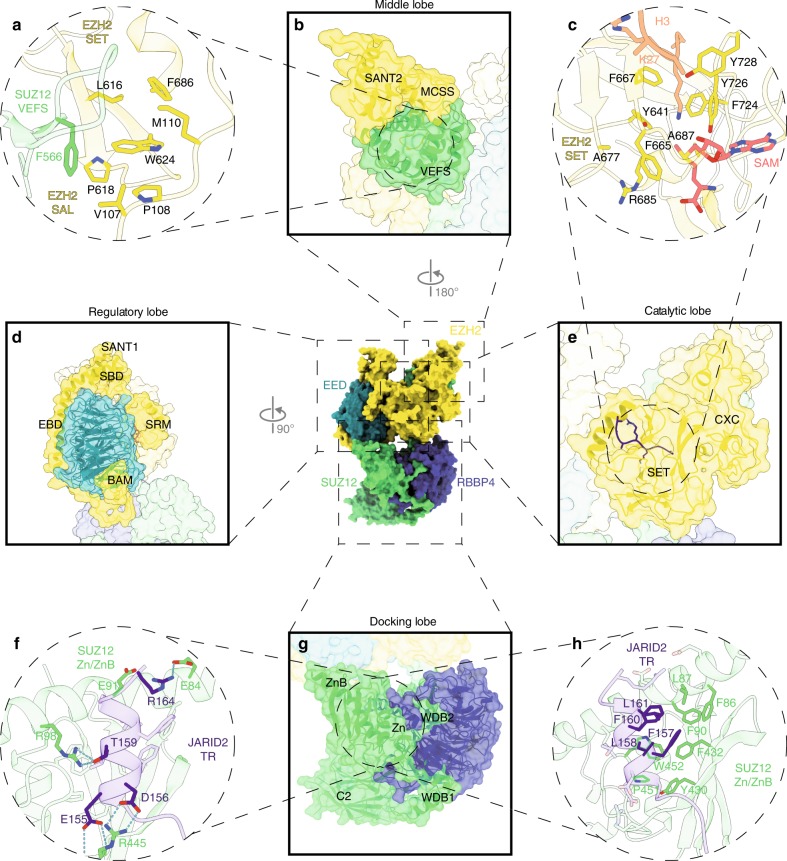
Four-lobed structure of the PRC2 core, comprising EZH2, SUZ12, EED and RBBP4 (PDB: 5WAI and 6C23). **a** The middle lobe is essential for PRC2 histone methyltransferase activity. The GWG motif of EZH2 (W624) is stabilised by a hydrophobic pocket at the interface between EZH2 (SAL/SET) and SUZ12 (VEFS) (PDB: 6C23). **b** The middle lobe extends to the back of the catalytic lobe (**e**), bridging it with the regulatory lobe (**d**). **c** PRC2’s HMT activity resides in the SET domain of EZH2. The lysine substrate (K27) is accommodated in a hydrophobic pocket that goes into the cofactor (SAM) binding pocket (PDB: 5TQR and 6C23). **d** EZH2 wraps around the EED WD propeller. Regulatory contacts occur at the open end of the propeller and are transmitted to the catalytic lobe (**e**) by the SRM domain of EZH2. **e** The catalytic lobe is formed by the SET and the CXC domains of EZH2. **f–h** The docking lobe is formed by the association of RBBP4/7 with SUZ12 N-terminus (**g**) and serves as a platform for the association of accessory factors. Binding of JARID2 TR domain to the SUZ12 Zn/ZnB pocket involves hydrogen bonds (**f**) as well as hydrophobic interactions (**h**) (PDB: 5WAI)

The association of the trimeric core (EZH2, SUZ12 and EED) with RBBP4 results in a stable, four-lobed structure (Fig. 1)^27,28^ that comes together to mediate HMT activity.

### The catalytic lobe

The C-terminal region of EZH2, comprising the CXC domain (a cysteine-rich region) and the SET domain, forms the catalytic lobe, in which the HMT activity of PRC2 resides (Fig. 1e). The active site presents two pockets in the SET domain: the first one is a highly hydrophobic channel (Y641, F667, F724, Y726 and Y728), which accommodates the long aliphatic chain of the lysine substrate (Fig. 1c). The end of this channel is connected to a second pocket, in which the cofactor S-adenosyl methionine (SAM) is positioned in an orientation that brings its methyl group in close proximity to the ε-amino group of the lysine. Residues that lie at the interface of these two pockets (e.g. Y641, A677 and A687) are crucial for catalysis, and their mutation results in changes in affinity for the substrate that are associated with gain-of-function phenotypes (as discussed below). The assembly of the trimeric core is essential for HMT activity: in isolation, EZH2 adopts an autoinhibited conformation, with the post-SET domain (the region C-terminal to the SET domain) folded upwards into the lysine-binding cleft, blocking the substrate from engaging the active site.^31–33^ This mechanism, which seems to be conserved in the H3K9 methyltransferase Suv39h2,^34^ might provide a ‘safety catch’ against spurious histone methylation.

### The regulatory lobe

The catalytic lobe is in close contact with the regulatory lobe, which is formed by the association of EED with the N-terminal domain of EZH2 (Fig. 1d). The long α-helix of the EED-binding domain (EBD) and the β-addition motif (BAM) of EZH2 wrap around the bottom (or closed end) and side, respectively, of the WD-repeat seven-bladed β-propeller of EED.^35,36^ This conformation is necessary to maintain EED in a stable position while leaving the opposite side of the propeller open for stimulatory ligand binding.^37^ The open end of the propeller faces the rest of the EZH2 N-terminal region. The SET activation loop (SAL) and the stimulation-responsive motif (SRM) of the EZH2 N-terminal region are responsible for the allosteric changes in the catalytic lobe upon trimethyl-peptide binding in the EED pocket. Indeed, although the trimeric core retains basal HMT activity, the recognition of the H3K27me3 mark further activates the complex resulting in an enhanced catalysis (discussed below). Finally, the loop around EED is closed by the association of the SANT1 domain of EZH2 with the distal part of EBD, named SANT1-binding domain (SBD).

### The middle lobe

The central part of EZH2 (comprising the MCSS and SANT2 domains) and the globular VEFS domain of SUZ12 are part of a bridging middle lobe (Fig. 1b). In particular, VEFS (which is packed between the regulatory and the catalytic modules) seals together the SAL domain and the GWG loop (W624) of the SET domain via a hydrophobic pocket (SUZ12 F566; EZH2 V107, P108, M110, L616, P618 and F686) (Fig. 1a), thereby stabilising the active site.^35^

### The docking lobe

Finally, the SUZ12 N-terminal region protrudes away from VEFS to form an extended fourth lobe that serves as a docking platform for factors that associate with PRC2 (Fig. 1g).^38,39^ RBBP4/7 is inserted into this lobe, its WD propeller tied by multiple interactions with the SUZ12 C2, WDB1 and WDB2 domains. The ‘cage’ built by SUZ12 around RBBP4/7 prevents the latter from binding many of its known nuclear interactors, including nucleosomes,^39^ thereby inhibiting its ability to sense active chromatin states. The functional relevance of incorporating an ‘inactive’ WD propeller into the PRC2 complex is still unclear.

Overall, spatial segregation of the docking lobe with respect to the rest of the complex reflects the functional separation between recruitment and catalysis, with the former mediated by accessory proteins, and the latter endowed into the other three lobes.

### PRC2-associated factors

**Fig. 2:**
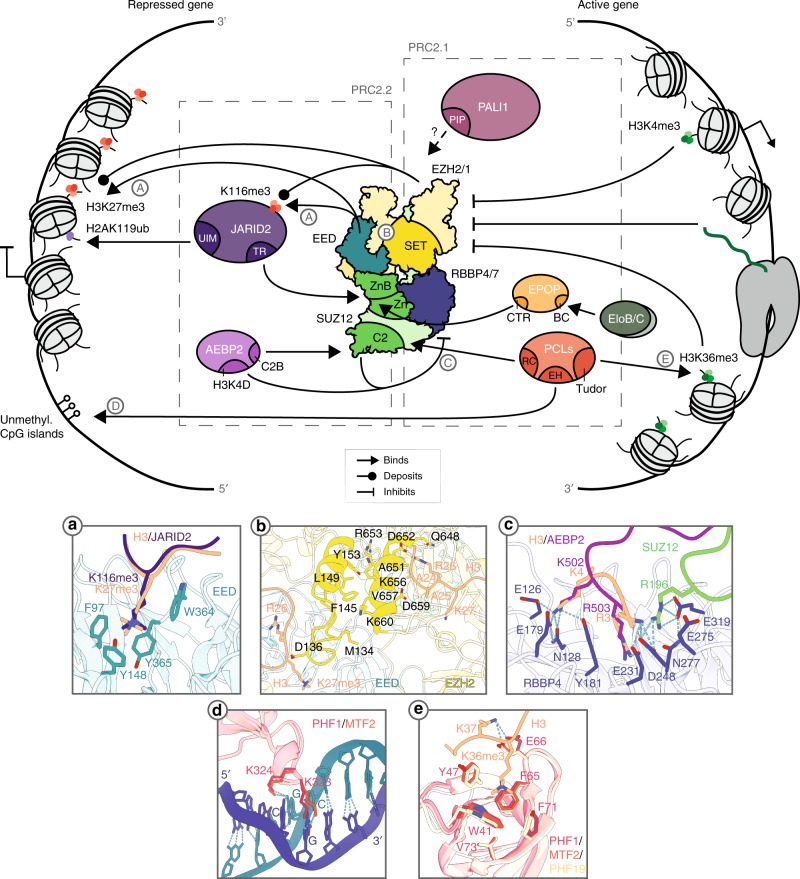
PRC2’s activity and recruitment depend on the chromatin context. **a** The EED aromatic cage of PRC2 is able to recognise both H3K27me3 and JARID2K116me3 (PDB: 3IIW and 6C23). **b** Recognition of the stimulatory ligands results in extensive interactions between the SAL, SRM and iSET domains, which results in the opening of the active site and enhanced histone methyltransferase activity (PDB: 5HYN). **c** Binding of the RBBP4 acidic pocket of unmodified H3K4 is inhibited in the context of the PRC2 complex (PDB: 2YBA and 5WAK). **d** PCL proteins specifically bind unmethylated CpG dinucleotides through their EH domain (PDB: 5XFQ and 5XFR). **e** The Tudor domain of PCL proteins is able to recognise the H3K36me3 mark (PDB: 4BD3, 5XFQ and 5XFR). Residues numbering in (**c**) and (**e**) is relative to PHF1

The interaction of facultative subunits with the PRC2 core, as well as the occurrence of mutual exclusivity, was based only on biochemical evidence until 2018, when two studies resolved the structure of the human PRC2 core complex in association with facultative subunits, in a first attempt at elucidating the structural basis for differential usage of associated factors, as well as their role in regulation of enzymatic activity^39,40^ These structures show that a non-canonical C2 domain in SUZ12 provides a binding platform for either the C2B domain of AEBP2 or the RC domain of PHF19 (Fig. 2). In parallel, the ZnB-Zn domain of SUZ12 functions as a docking site for the C-terminal region of EPOP as well as the transrepression (TR) domain of JARID2 (Fig. 1f, h**;** Fig. 2).^39^ These findings provided the first structural evidence for the previously observed mutual exclusiveness of the binding to PRC2 of EPOP/PCL proteins and AEBP2/JARID2. However, they still did not rule out the possibility that hybrid complexes might form containing either AEBP2–EPOP or PCL–JARID2. Indeed, the appearance of a hybrid MTF2–JARID2 complex was observed upon AEBP2 depletion in mouse ESCs, suggesting that further protein–protein interactions help dictate which pair of subunits interacts with the core complex.^14^

Although these structures provide crucial insight into PRC2 holocomplex formation, many PRC2-associated factors have yet to be characterised in such detail, such as the ELOB and ELOC heterodimer (also termed TCEB2 and TCEB1, respectively; hereafter referred to as ELOBC), and the PALI1/2 proteins. However, some biochemical evidence provides preliminary insights into how these factors associate both with the core complex and other accessory proteins. In mouse ESCs, ELOBC associates with PRC2 on chromatin^28,29^ in an interaction mediated by EPOP (depletion of EPOP disrupts the association of ELOBC with PRC2 and its subsequent binding on chromatin). Binding of ELOBC to EPOP (and, consequently, to PRC2) is mediated by the BC box motif, a classical ELOBC binding motif, on the N-terminus of EPOP. Mutation of a crucial leucine residue (L40) in this motif impairs the association of ELOBC with EPOP and PRC2.^28,29^ The interaction of EPOP with ELOBC is crucial for proliferation of several human cancer cell lines. Indeed, depletion of EPOP leads to a decrease in their proliferative capacity. This phenotype is dependent on EPOP interaction with ELOBC as only the wild-type version of the EPOP protein (but not the L40 mutant) is able to rescue cancer cells proliferation.^29^

PALI1 and 2 are vertebrate-specific subunits of the PRC2.1 complex, which associate with PRC2 through their highly conserved C-terminal PALI-interaction with PRC2 (PIP) domain (Fig. 2). Structural information about these proteins is still lacking, but two tryptophan residues (W1125 and W1186 in PALI1) conserved in both PALI proteins have been found to play an essential role in PRC2 binding, as the concomitant mutation of both residues abolishes the association of the PIP domain with PRC2 in vitro.^30^ Although this biochemical evidence provides crucial information about how these factors associate with PRC2, the exact molecular functions of EPOP, ELOBC or PALI1/2 within PRC2 so far remain unknown.

## Functional interplay between PRC2 and chromatin

PRC2 mediates gene repression through direct interaction with its target genes and deposition of the repressive H3K27me3 mark. The ability of PRC2 to identify its target genes and correctly perform its enzymatic activity relies on a complex but tightly regulated interaction with different elements of the chromatin environment. This is mainly facilitated by the many sub-stoichiometric associated factors that provide PRC2 with the capability to bind DNA, nucleosomes, histones and RNA.

### DNA and nucleosomes

In *Drosophila melanogaster*, PcG proteins are recruited to their target genes via Polycomb response elements (PREs), which are *cis*-regulatory DNA sequences that provide a platform for Polycomb subunit binding and subsequent gene repression.^41,42^ Although Polycomb proteins are well conserved between flies and mammals (reviewed in ^43^), no PRE-like specific DNA sequences have been identified to date in mammals.^44^

Of the core components of PRC2, both EZH2 and SUZ12 possess zinc finger domains, which could potentially mediate an interaction with DNA. However, rather than playing a major role in targeting PRC2 to chromatin, binding of the core components of the complex to DNA seems to stabilise the interaction of PRC2 with the nucleosome template and thereby facilitate catalysis; specifically, the EZH2 CXC Zn-binding clusters contact the DNA exiting from the substrate nucleosome. Additionally, the EZH2 SBD domain contacts the DNA minor groove in the H3K27me3-marked nucleosome, after it has been bound by EED. Concurrently, EED might also contact the DNA of the modified nucleosome, through an unstructured, positively charged N-terminal stretch.^45^ Stable interaction with the chromatin is indeed key for PRC2 stimulation: HMT activity is enhanced on di-/oligonucleosomes as compared with mononucleosomes, especially for linker DNA with lengths of 35–40 bp, allowing for engagement of both the stimulating and the substrate lysine residues.^45,46^ Interestingly, this feature seems to be specific for EZH2–PRC2, as EZH1–PRC2 does not display such a preference for its substrate.^46^

AEBP2 and JARID2 contain three and one zinc finger domains, respectively, with JARID2 also harbouring an AT-rich-interacting domain (ARID). Both proteins display DNA-binding ability in vitro^47,48^ and were therefore the first candidates to be considered for facilitating DNA-mediated recruitment of PRC2. AEBP2 was shown to have a binding preference for a highly degenerate consensus sequence with a bipartite structure (CTT(N)_15–23_cagGCC),^49^ suggesting that the recognition of this motif could provide a targeting mechanism for PRC2. In addition, in vitro biochemical assays showed that the presence of AEBP2 increases the capacity of PRC2 to bind nucleosomes, through its KR motif (a lysine/arginine-rich positively charged patch), resulting in enhanced HMT activity.^46^ Nonetheless, AEBP2 does not seem to be essential for PRC2 recruitment in vivo, as depletion of AEBP2 in mouse ESCs does not affect PRC2 occupancy on chromatin and even leads to a slight increase in H3K27me3 levels.^14^ For JARID2, in vitro experiments have demonstrated its ability to bind DNA, with a slight bias for GC-rich elements. This binding is mediated by the C-terminal region of JARID2, potentially through its C5HC2 zinc finger.^50^ Independent of its DNA-binding ability, JARID2 can also mediate the interaction with nucleosomes, resulting in enhanced PRC2 activity. This interaction is mediated by a region with no annotated structure, which spans residues 349–450.^51^ In line with these observations, evidence has shown that PRC2.2—but not PRC2.1—is able to bind nucleosomes in vitro.^39^ In agreement with a role for JARID2 in mediating PRC2 recruitment to chromatin loci, depletion of JARID2 reduces PRC2 levels at specific target genes.^50,52–54^

Notably, JARID2 is mutated and/or deleted in various types of leukaemia where PRC2 plays an onco-suppressor role.^55,56^ This suggests that JARID2-mediated PRC2 recruitment is essential for its tumour-suppressive functions.

PCL proteins are essential for PRC2 recruitment, as their depletion in mouse ESCs leads to a strong reduction in the levels of PRC2 on chromatin.^57–59^ In 2017, Li and colleagues^59^ resolved the crystal structure of human N-terminal PHF1, which contains Tudor, PHD1 and PHD2 domains as well as a newly identified extended homologous (EH) domain. The EH domain folds into three α-helices and a curved three-stranded β-sheet. The W1 loop of the winged helix structure within the EH domain (EH_WH_) binds to unmethylated CpG DNA motifs, but not to GpC or AT-rich elements. Binding specificity is provided by the first two lysine residues of the W1 loop (K323 and K324 in PHF1), which form extensive contacts with both cytosine and guanine nucleotides (Fig. 2d), and their mutation to alanine abolishes the DNA-binding ability of all three PCL proteins.^59^ This interaction with DNA increases the residence time of PRC2 on nucleosomes and on naked DNA, leading to its increased catalytic activity.^60^ These results provide a potential explanation for the observed correlation between PRC2-occupied sites and unmethylated CpGs observed in vivo.^8,61^ However, a large fraction of unmethylated CpG islands remains unbound by PRC2 in vivo, suggesting that additional features might be required for target specificity. Consistent with this, a 2018 report showed that specific DNA helical shape characteristics (a wider minor groove and a decreased propeller and helix twist) are required for MTF2 to bind to unmethylated CpG motifs,^62^ therefore narrowing down the potential target regions. These observations point to a more degenerate definition of vertebrate PREs that would be defined by DNA helical shape rather than specific primary sequence.

### Histone post-translational modifications

Another crucial aspect of PRC2 regulation and recruitment on chromatin comes from its direct interaction with many post-translation modifications that are present on histone tails. Indeed, PRC2 activity strongly depends on the epigenetic state of its chromatin environment.

On the one hand, recognition of PRC2-deposited H3K27me3 products by EED results in catalytic stimulation of the complex: the open end of the EED WD-propeller is oriented towards the side of the catalytic lobe, thereby exposing its aromatic cage in this direction. Following the engagement of H3K27me3 with the aromatic cage (F97, Y148, Y365 and W364; Fig. 2a), the SRM domain of EZH2 makes extensive interactions with both EED and the trimethyl-peptide, passing from a disordered unstructured conformation to a fully structured α-helix shape. This conformation can now contact the iSET domain, which undergoes a counterclockwise rotation of 20°, leading to the opening of the SET substrate-binding cleft (Fig. 2b).^35,37,63,64^ These dynamics result in 4–6-fold enhanced substrate binding^35^ and 3–7-fold increased catalytic activity.^37,65^ Interestingly, H3K27me3 is not the only epigenetic mark that can be recognised by EED, as other repressive chromatin marks (e.g. H1K26me3, H3K9me3, H4K20me3)—but not active marks (e.g. H3K4me3, H3K36me3, H3K79me3)—are bound with comparable affinity. However, H3K27me3 is the only mark that is able to elicit PRC2 catalytic stimulation.^37,65^ Although it is tempting to speculate that EED-mediated recognition of repressive histone marks could serve as a mechanism for recruitment of PRC2 to silenced loci, a large body of evidence has shown that stimulation of PRC2 catalytic activity and its recruitment on chromatin are completely uncoupled.^64,66–71^ Rather, this mechanism accounts for a positive-feedback loop: upon PRC2 deposition of the H3K27me3 mark through its basal level of activity, methylation of the neighbouring nucleosome is favoured, resulting in spreading of the histone mark along the linear chromatin fibre as well as along the regions that are in spatial proximity.^72^

In addition to this, PRC2 is also able to self-stimulate via trimethylation of JARID2 at lysine 116 (JARID2K116me3) (Fig. 2a). This modification mimics that of H3K27me3 and is able to elicit the same structural rearrangement,^73^ but the physiological relevance of this mechanism is not yet clear. However, as JARID2K116me3 activates PRC2 catalysis regardless of the presence of the H3K27me3 mark, this mechanism could account for the deposition of the mark on unmodified nucleosomes. In other words, the incorporation of JARID2 in the complex could serve as a mechanism to confer PRC2 with a pioneering function that can initiate silencing.

Besides its own marks, PRC2 also recognises marks set by PRC1. Indeed, although H3K27me3 deposited by PRC2 is known to provide a docking site for PRC1 through its recognition by CBX proteins,^74^ this inter-complex crosstalk appears to be bidirectional, as PRC2 can bind the H2AK119ub mark deposited by PRC1.^75^ This binding is specifically mediated by an ubiquitin-interaction motif (UIM) on the N-terminus of JARID2 and is essential for PRC2 recruitment to H2Aub119-marked regions (Fig. 2).^76^ This creates a positive-feedback loop between PRC2 and PRC1 that keeps genes from exiting this repressed state.

On the other hand, however, histone post-translation modifications associated with an active transcriptional state (e.g. H3K4me3 and H3K36me2/3) have the opposite effect, strongly inhibiting PRC2 di- and tri-methylation activity.^77,78^ Initial studies focusing on the role of the nucleosome-binding proteins RBBP4/7^79–81^ revealed that the ability of these proteins to bind histone tails is inhibited by the presence of H3K4me3,^82^ leading to speculation that these proteins could serve as a sensor for active chromatin states. Although appealing, this model was soon rejected, as H3K4me3-dependent PRC2 inhibition is independent of RBBP4 inclusion in the complex: a minimal EZH2–EED–SUZ12(VEFS) complex retains both HMT activity and inhibition by H3K4me3/H3K36me2/3.^77^ Two studies published in 2018 have helped to explain how RBBP4 progressively loses its nucleosome-binding capacity in the context of the human PRC2 complex: distinct residues from SUZ12 (C2 R196) and AEBP2 (H3K4D, K502 and R503) directly compete with the H3 tail for binding to the RBBP4/7 acidic pocket (comprising E126, N128, E179, Y181, E231, D248, E275, N277 and E319) (Fig. 2c). Moreover, PRC2 appears to engage dinucleosomes in an orientation that is incompatible with the interaction of RBBP4/7 with the H3 tail.^45^ Finally, SUZ12 (WDB1, WDB2) wrapping around RBBP4/7 eventually imposes steric hindrance to the interactions of RBBP4/7 with histone H4.^39^

An alternative hypothesis has therefore been put forward by two reports showing that EZH2 is able to sense the modification state of both H3K36 and H4K16/20 via two independent pockets located in its CXC/SET and SANT1 domains, respectively.^83,84^ Although the functional relevance of H4 binding is still unclear, Jani and colleagues^83^ have shown that unmodified H3K36 (but not H3K36me2/3) can increase the catalytic activity of EZH2, providing a rationale for the lower PRC2 activity in active chromatin environments.

As well as the PRC2 core components, the PCL family of proteins has long been known to interact directly with the H3K36me2/3 mark. PHF1, PHF19 and MTF2 can all bind the H3K36me2/3 mark through their aromatic cage (W41, Y47, F65, F71 and V73 in human PHF1) located in their highly conserved Tudor domain (Fig. 2e).^57–59,85–87^ Interactions with these histone marks are abolished when any of these residues is mutated to an alanine.^57,85^ The significance of this interaction for PRC2 recruitment remains unclear, considering the limited overlap between the genomic localisation of H3K36me3 and H3K27me3 on chromatin in mouse ESCs.^57^ One of the proposed mechanisms is that PCL-containing PRC2 transiently binds to H3K36me3, together with a H3K36me2/3-specific demethylase (such as KDM2B or NO66), in order to establish *de novo* H3K27me3 domains and to impose gene silencing during differentiation.^57,58^ Thus, while histone post-translation modifications associated with active transcription could mediate negative feedback on EZH2 resulting in inhibition of PRC2 activity, an intriguing possibility is that incorporation of PCL proteins into PRC2.1 confers on the complex the ability to initiate the silencing of actively transcribed regions.

However, many genomic regions in mouse ESCs show co-localisation of the H3K36me2 and H3K27me2 marks: in this scenario, the H3K36me2 mark, deposited by NSD1, prevents the spreading of H3K27me3.^88^ Notably, H3K36me2/3 decorated regions seem to be devoid of PRC2,^88,89^ suggesting that H3K27me2 deposition at these loci is the result of a transient interaction. Whether PCL proteins play a role in this mechanism is not clear yet.

### RNA

Along with the many chromatin features that regulate PRC2 recruitment and activity, other studies suggest that RNA might play a role in both of these two processes: despite the lack of a known RNA-binding domain, PRC2 binds RNA in vivo and in vitro in a non-sequence-specific manner, although it does show a preference for GC-rich sequences and G-quadruplexes.^90–92^ Moreover, direct binding to RNA results in inhibition of PRC2 HMT activity both in vitro^90,93^ and in vivo,^94–96^ probably due to competition with chromatin binding (Fig. 2)^95,97^ (for a comprehensive discussion please refer to^98,99^).

However, the molecular mechanisms of binding and of the resulting inhibition are not yet clear. To address these questions, Zhang and colleagues^96^ used a targeted proteomic identification of RNA-binding regions (RBR-ID) approach to map, at high resolution, the interactions within the nucleus between PRC2 components and RNA. Their results showed that binding involves neither a single subunit of the complex nor discrete RNA-binding domains within them but, rather, dispersed amino acid patches on the exposed surface of the complex;^96^ this is in line with previous in vitro observations by ﻿hydrogen deuterium exchange mass spectrometry.^100^ Interestingly, RNA interactions were observed for all PRC2 subunits (that is, all core and accessory factors belonging to PRC2.1 and PRC2.2). However, the most consistent points of interaction were at regions of EZH2 at the interface with EED (namely, the SBD, EBD, SRM and iSET domains), leading the authors to speculate that inhibition of PRC2 HMT activity by RNA could be, at least partially, due to interference with transduction of the stimulatory signal.^96^ These observations suggest that the inhibition of PRC2 activity in active chromatin environments is mediated not only at the epigenetic level (through negative regulation by H3K4me3 and H3K36me2/3) but also by RNA itself, as the final output of actively transcribed regions.

## Mutations in PRC2 components and their roles in cancer

Early observations demonstrated that EZH2 was overexpressed in prostate tumours and played a role in the progression of this cancer type.^101^ Since then, many reports have shown that other PRC2 factors are subject to deletions, mutations, translocations and/or dysregulation in different cancer types. In the majority of cancer types, high expression of PRC2 factors correlates with sustained proliferation of cancer cells.^102,103^ However, whether this reflects a causative role for PRC2 in tumorigenesis is difficult to assess. In some cases, PRC2 has been shown to repress key tumour-suppressor genes, therefore directly promoting tumour progression.^18,104,105^ Accordingly, a lot of effort is being put in developing targeting strategies to inhibit the activity of PRC2 in order to treat various cancer types (for a comprehensive review on PRC2 inhibition strategies see ^19,106,107^).

PRC2 genes, both core and associated factors, are mostly mutated in haematological malignancies. This is in line with their role in haematopoiesis, where they regulate crucial processes such as hematopoietic stem cells self-renewal and B-cells germinal centre formation.^19^ In haematological malignancies, contrary to most other cancer types, PRC2 can exhibit both oncogenic and tumour-suppressor functions, depending on the cellular context.^19,105^

### SET domain loss-of-function mutations

**Fig. 3:**
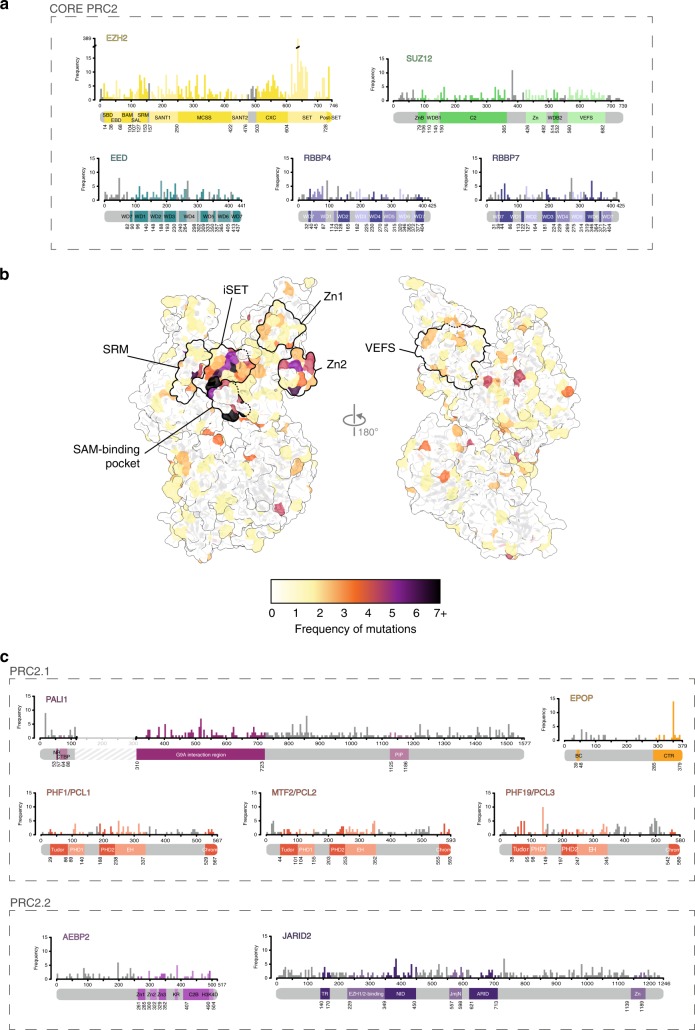
The PRC2 cancer mutational landscape. **a** Missense mutation frequency mapped on the linear structure of PRC2 core components (bin = 5 residues). **b** Structural mapping of missense mutations of PRC2 genes in cancer. Numbers were obtained from the Catalogue Of Somatic Mutations In Cancer (COSMIC) database^108^ and mapped on a composite structural model of PRC2, comprising core components with the addition of JARID2 TR domain and AEBP2 C-terminus (PDB: 5WAI and 6C23). **c** Missense mutation frequency mapped on the linear structure of PRC2-associated factors (bin = 5 residues)

Among all PRC2 factors, EZH2 displays the highest rate of mutations (data from the COSMIC database)^108^ (Fig. 3a–c), in line with its essential role as the catalytic subunit of the complex. Indeed, most of the mutations in this protein occur in the CXC and SET domains, which are required for HMT activity. In particular, in the SET domain, hotspots of mutations are localised on residues that form part of the active site, binding both the substrate and the SAM cofactor (Fig. 3a, b).

As previously discussed, the lysine substrate is accommodated in an aromatic cage (Fig. 1c), and mutations of the hydrophobic residues involved in this structure (F665C/L/S, F667L, Y726C/D/N/H and Y728H) are mostly found in myeloid malignancies, mainly in acute myeloid leukaemia (AML), chronic myeloid leukaemia (CML) and myelodysplastic syndrome (MDS).^109–116^ Disruption of one of these residues could result in impaired substrate recognition and, possibly, loss of methyltransferase activity, as demonstrated for the Y726D mutant.^113^ The R685 residue, which lies just below the bottom of the hydrophobic pocket, seems to be essential for the correct orientation of F665, and its mutation to cytosine abolishes methyltransferase activity in vitro.^113,117^ Indeed, this amino acid is found mutated (R685C/H) in AML, early T-cell precursor acute lymphoblastic leukaemia (ETP-ALL), MDS and myelofibrosis.^113,115,117–124^

iSET is also responsible for stabilisation of the H3 substrate, as it is sensitive to the activating signal coming from the EED–trimethyl-peptide–SRM axis. Specifically, the α-helix of iSET shapes the channel in which the H3 tail lies and is essential for enhancing PRC2 activity upon stimulus (Fig. 2b). Mutations in many of the residues that face the H3 tail (e.g. Q648, A651, D652, G655 and D659) are found in AML, MDS and T-cell acute lymphoblastic leukaemia (T-ALL).^110,111,113,115,116,125–130^ These mutations can either cause steric hindrance to the H3 tail (e.g. G655R) or abolish interactions between the H3 backbone and the iSET (for instance, D659V/A/G impairs the stabilisation of the two alanine residues preceding the lysine substrate). However, to our knowledge, the impact of these mutations on the HMT activity or stimulation of PRC2 has not been assessed.

With respect to the SAM-binding cleft, residues forming the walls of this channel (V621, N688, H689 and S690) are essential, and their relevance is underscored by their relatively high frequency of mutation in AML, CML, ETP-ALL and MDS^110–113,115,117,122,130–134^ (Fig. 3b). In particular, the mutations V621M and H689R abolish the HMT activity of PRC2, probably by impairing SAM binding and abrogating the deposition of H3K27me3 on chromatin.^117,135^

### SET domain gain-of-function mutations

Mutations in Y641 of EZH2 have been observed to occur at a high frequency (in around 25% of patients) in patients with follicular lymphoma (FL) and diffuse large B-cell lymphoma (DLBCLs),^113,136–149^ as well as in those with melanoma^151–152^ and Ewing sarcoma.^153,154^ Mutations of this residue are thought to be an early driving event in cancer progression, as expression of the EZH2 Y641F mutant in mouse B-cells and melanocytes leads to the formation of lymphoma and melanoma, respectively.^137,155^ At the biochemical level, the EZH2 Y641F mutant shows an increased ability to modify dimethyl substrates yet is almost unable to act on nucleosomes with unmethylated or monomethylated H3K27.^64,156–158^ Molecularly, Y641 is positioned at the interface between the substrate lysine and the SAM cofactor, with the potential to form hydrogen bonds with the unmethylated lysine (Fig. 1c). Mutation of Y641 is thought to enlarge the binding channel, rendering the mutant form more permissive to H3K27me2 engagement but preventing this residue from interacting with the unmethylated substrate.^144,159^ Although initially interpreted as a loss-of-function mutation, it was later discovered that this mutation can act synergistically with the wild-type form of EZH2, as the latter is more proficient on H3K27me0/1 substrates and can provide the perfect substrate (H3K27me2) for the Y641 mutant form, resulting in overall hyper-trimethylation.^137,157,158,160,161^ Indeed, mutations in Y641 are exclusively found in heterozygosity. How this excess of H3K27me3 confers tumorigenicity is not well understood, but evidence points to a role for this mark in mediating the silencing of multiple tumour suppressors through changes in promoter-enhancer contacts within chromatin compartments.^162^

Two other gain-of-function mutations, A677G and A687V, are present in around 1–2% of patients with FL or DLBCL.^142–144,156,163^ The A677G mutation enlarges the lysine-binding pocket of EZH2 without affecting the interaction of Y641 with the unmodified substrate, therefore resulting in highly efficient catalysis on all methylation states and eventually leading to aberrantly high levels of H3K27me3 and lower levels of H3K27me2.^35,156^ On the other hand, A687V increases the efficiency of EZH2 for monomethyl substrates, by weakening the interaction with a water molecule that needs to be displaced for demethylation to happen. This results in a more balanced substrate preference with respect to wild-type EZH2, thereby increasing overall levels of H3K27me3 without affecting those of H3K27me2.^164^ Presumably, the resulting aberrantly high levels of H3K27me3 are responsible for the tumorigenicity of these EZH2 mutants.

### CXC domain mutations

The two zinc-binding clusters inside the CXC domain (Zn3C9 and Zn3C8H1) are placed right on top of the active site and are involved in contacting DNA in the substrate nucleosome.^45^ These clusters accumulate a high frequency of mutations, probably leading to loss-of-function mutant isoforms that are not able to stabilise the interaction of PRC2 with the nucleosome. So far, only the C571W mutation has been found in homozygosis in myelodysplasia patients. EZH2 proteins bearing this mutation show complete loss of PRC2 HMT activity and can partially destabilise the integrity of the PRC2 complex.^113^

### Stimulatory axis mutations (EED–SAL–SRM)

The SAL and SRM domains of EZH2 are responsible for transducing the stimulatory signal from the recognition of the H3K27me3 or JARID2K116me3 mark by EED to the iSET motif (Fig. 2b). Almost all residues involved in this process can be mutated in different types of tumour. Among these, major hotspots are represented by P132 and F145, which have been found to be mutated in patients with myelofibrosis, MDS or T-ALL^111,113,116,120,121,126,165^ (Fig. 3a, b). Interestingly, mutation of these residues specifically affects PRC2 stimulation but not its basal activity, complex stability or recruitment to chromatin. Moreover, mouse ESCs expressing these EZH2 mutant forms show a dramatic reduction of H3K27me2/3 levels and an impaired pluripotency capacity,^64^ demonstrating that stimulation of PRC2 HMT activity is key for its correct functioning in the nucleus. In line with these results, mutations in EED that affect its interaction with the SAL–SRM module (namely, S259F and R302G) lead to the very same stimulation-deficient phenotype.^64^ S259F and R302G mutations have been observed in T-ALL and myelofibrosis patients, respectively.^123,132^

### VEFS domain mutations

The VEFS domain has a moderately high frequency of mutations, with a diffuse mutational pattern rather than specific hotspots (Fig. 3a, b). Many of these mutations have been experimentally validated in vitro, and indicate that even single amino acid substitutions in the VEFS domain can result in a dramatic decrease in the HMT activity of PRC2. This is the case for F603L, D605V and E610G, which have been found in patients with chronic myelomonocytic leukaemia (CMML), myelofibrosis or B-ALL.^166,167^ In addition to these mutations, other mutations such as W591C/R and N618Y have been observed in osteosarcoma and T-ALL^130,168^ and could have deleterious effects: mutations of the homologous residues in *D. melanogaster* Su(z)12 result in decreased HMT activity and impaired complex assembly.^169^ Interestingly, N618 forms H bonds with the amine and carboxyl groups of Y292 in the CXC domain, suggesting that mutation to a tyrosine residue would disrupt this interaction and thereby destabilise SUZ12’s association with EZH2.

### Other hotspots

Apart from the aforementioned hotspots, only a few other residues in most of the PRC2 proteins show a higher frequency of mutation with respect to the rest. However, the functional impact of most of these mutations has not yet been addressed, in some cases due to the lack of a resolved structure. This is the case for EZH2 E740K/Q in both solid tumours and leukaemias^108,115,126,146,170,171^ (Fig. 3a). This residue lies in the post-SET region and its mutation could potentially affect the autoinhibition mechanism of EZH2 (see above), resulting in aberrant H3K27me3 deposition and consequently transcriptional deregulation. EPOP A350V/P mutations, which have been observed in lung and thyroid tumours,^172^ reside in the C-terminal region of EPOP, which is essential for its binding to PRC2 (Fig. 3c). AEBP2 A198E and R388Q have been observed in kidney and oesophagus tumours, respectively.^173,174^ The latter mutation, located in the positively charged patch of the KR domain, potentially affects the interaction of AEBP2 with nucleosomes and, consequently, PRC2 stimulation^46^ (Fig. 3c). Finally, the SUZ12 mutations E383G/K/V have been observed in oesophageal squamous cell carcinomas (SCC);^174^ the EED mutations R52C/H/L/P in a variety of solid tumours^108,146^ (Fig. 3a); and the PHF19 mutation D136A in thyroid neoplasms and upper aerodigestive tract SCC^108,174^ (Fig. 3c). The consequences of these latter mutations on PRC2 assembly and activity are difficult to predict as they do not involve residues of known function.

## Conclusion

The PRC2 complex is a tightly regulated machine that governs the correct spatial and temporal expression of key developmental regulators via its function as a transcriptional repressor. Its activity is regulated via interactions with both facultative subunits and its chromatin environment. In general, the interaction of PRC2 with chromatin appears to be a way to safeguard the different chromatin states. Indeed, PRC2 is able to perpetuate a repressive state in a number of ways: first, by binding its own H3K27me3 mark; second, by binding the H2A119ub mark deposited by PRC1; and, third, by trimethylating and binding to JARID2K116. All of these interactions increase PRC2 activity, either by increasing residence time on chromatin (by PHF1, for example^60^) or stabilising its interaction with nucleosomes through AEBP2 and JARID2.^46,51^ By contrast, an active chromatin state is maintained by inhibition of PRC2 activity through the inhibition of the core complex by H3K4me3 and H3K36me3, and by PRC2 binding to RNA, which impedes chromatin binding and/or transduction of the stimulatory signal. In other words, both chromatin states are able to maintain a positive-feedback loop through, in part, inhibition or activation of the PRC2 complex. However, in some cases, maintenance of a chromatin state can be challenged. For instance, both AEBP2 and JARID2 can mimic histone tails through their binding to PRC2. This competition between PRC2 subunits and histone tails could be a mechanism by which PRC2 autoregulates its activity to override the epigenetic context of the targeted chromatin.

Evidence points towards the idea that the catalytic modulation of human PRC2 is structurally uncoupled from its recruitment to chromatin. The minimal complex of EZH2–EED–VEFS(SUZ12) is sufficient to mediate enzymatic activity and modulate it by sensing the chromatin state, as is reflected by the higher incidence of missense mutations in the domains that mediate the catalysis and stimulation of methyltransferase activity.

Interestingly, the ability of core components to interact with chromatin features seems to be limited to modulation of the catalysis rather than recruitment to target regions. This observation is in line with the emerging view that differential recruitment of PRC2 during distinct developmental stages is achieved by incorporating accessory factors that are dynamically expressed during development. Cell-type-specific expression and partial redundancy of accessory subunits could therefore account for their lower mutation rates observed in cancer In line with this, it is worth noting that depletion of PRC2 accessory subunits only slightly affects genome-wide levels of H3K27me3. This suggests that, once the pattern is established, the core complex alone (without accessory factors) is able to maintain it, potentially through the positive-feedback mediated by the EED–SRM–iSET axis. Therefore, mutations/dysregulation of core components might be necessary to induce changes in the methylation pattern, as observed during tumorigenesis. While structural studies have greatly deepened our understanding of the function and regulation of PRC2, many unanswered questions relating to PRC2 and its role in controlling gene expression remain to be addressed. For instance, what is the role of the recently characterised associated subunits (e.g. EPOP, PALI1/2, ELONGIN B/C), for which structures with the complete core complex are still lacking? How much of our knowledge about EZH2 function and structure applies to EZH1, considering their differential response to stimulatory elements as well as their distinct mutational landscape? Furthermore, what is the impact of those mutation hotspots for which we have neither structural nor functional details? Answering these questions will help us to better understand both the physiological and the cancer-associated functions of PRC2.

## Data Availability

Not applicable.
